# Chronic lymphocytic leukemia cells from ibrutinib treated patients are sensitive to Axl receptor tyrosine kinase inhibitor therapy

**DOI:** 10.18632/oncotarget.26444

**Published:** 2018-12-14

**Authors:** Sutapa Sinha, Justin C. Boysen, Kari G. Chaffee, Brian F. Kabat, Susan L. Slager, Sameer A. Parikh, Charla R. Secreto, Tim Call, Tait D. Shanafelt, Jose F. Leis, Steven L. Warner, David J. Bearss, Asish K. Ghosh, Neil E. Kay

**Affiliations:** ^1^ Division of Hematology and Mayo Clinic, Rochester, MN, USA; ^2^ Department of Health Sciences Research, Mayo Clinic, Rochester, MN, USA; ^3^ Department of Medicine-Hematology, Stanford School of Medicine, Stanford, CA, USA; ^4^ Department of Hematology and Oncology, Mayo Clinic, Scottsdale, AZ, USA; ^5^ Tolero Pharmaceuticals, Inc., Lehi, UT, USA; ^6^ Stephenson Cancer Center and Department of Pathology, The University of Oklahoma Health Sciences Center, Oklahoma City, OK, USA

**Keywords:** AXL, TP-0903, Ibrutinib, CLL, apoptosis

## Abstract

Earlier we have shown the expression of a constitutively active receptor tyrosine kinase Axl in CLL B-cells from previously untreated CLL patients, and that Axl inhibitor TP-0903 induces robust leukemic B-cell death. To explore whether Axl is an effective target in relapsed/refractory CLL patients, we analyzed CLL B-cells obtained from CLL patients on ibrutinib therapy. Ibrutinib-exposed CLL B-cells were treated with increasing doses (0.01- 0.50μM) of a new formulation of high-affinity Axl inhibitor, TP-0903 (tartrate salt), for 24 hours and LD_50_ doses were determined. Sensitivity of CLL B-cells was compared with known prognostic factors and effect of TP-0903 was also evaluated on Axl signaling pathway in CLL B-cells from this cohort. We detected sustained overexpression of Axl in CLL B-cells from CLL patients on ibrutinib treatment, suggests targeting Axl could be a promising strategy to overcome drug resistance and killing of CLL B-cells in these patients. We found that CLL B-cells from sixty-nine percent of relapsed CLL patients actively on ibrutinib therapy were found to be highly sensitive to TP-0903 with induction of apoptosis at nanomolar doses (≤0.50 μM). TP-0903 treatment effectively inhibited Axl phosphorylation and reduced expression levels of anti-apoptotic proteins (Mcl-1, XIAP) in ibrutinib exposed CLL B-cells. In total, our *in vitro* preclinical studies showing that TP-0903 is very effective at inducing apoptosis in CLL B-cells obtained from ibrutinib-exposed patients supports further testing of this drug in relapsed/refractory CLL.

## INTRODUCTION

In 2018, there were as many as 21,000 new CLL cases diagnosed [1]. Based on iwCLL 2008 criteria [2], current approaches for upfront therapy for progressive CLL patients are chemoimmunotherapy (CIT) or the Btk signal inhibitor, ibrutinib. Despite the efficacy of CIT and the high overall responses with these two approaches, patients still experience relapse or become refractory. The Food and Drug Administration (FDA) approved options for relapsed disease from CIT includes ibrutinib or idelalisib with rituximab and more recently venetoclax. However, the complete response (CR) rates are low with these agents, and most responses are partial [3]. There are also evolving issues for use of these approved signal inhibitors including drug resistance and drug intolerance [4–7]. Importantly for ibrutinib, if it is discontinued for reasons including toxicity or refractory disease, there can be very rapid progression of the disease, and alternate therapeutic plans need to be in place for these patients [7]. The only FDA approved novel inhibitor strategies that may be of assistance when ibrutinib is unable to be used are idelalisib with rituximab or venetoclax [8]. However, significant toxicities have been reported for the regimen of idelalisib/rituximab with excess infectious deaths [3] and risk of immune based hepatic toxicity for CLL patients treated upfront, and the increased risk of tumor lysis syndrome with venetoclax. Off-target effects for both ibrutinib and idelalisib include low-grade, chronic adverse events (i.e., rash, arthralgia, atrial fibrillation, hemorrhages, and diarrhea) which are very troublesome for patients [9]. Venetoclax while showing stronger efficacy with more complete responses in relapsed/refractory CLL has issues in regard to tumor lysis syndrome and often initially requires hospitalization.

Alternate drug therapies are needed which have enhanced specificity for their target and which can be used in the face of resistance or prior exposure to the currently approved signal inhibitors. To this end, our group has discovered a novel membrane target in the ubiquitous presence of Axl on CLL B-cells. Axl is a member of the TAM receptor tyrosine kinase family that also includes Tyro3 and Mer [10, 11] and overexpression of Axl is reported in several human cancers [12–15]. Axl activation and signaling have been implicated in multiple cellular responses including cell survival, proliferation, migration, adhesion and also in drug resistance. Axl is over-expressed and activated in CLL B-cells compared to normal B-cells, and we have shown that targeting Axl using a drug designated as TP-0903 (free base) induces robust apoptosis at nanomolar doses in CLL B-cells from previously untreated CLL patients [16]. This drug is most selective for Axl RTK inhibition but does also modify, albeit with less selectivity, other cellular targets. Given the efficacy of this Axl inhibitor in CLL B-cells, we became interested in further exploring its preclinical activity in CLL in particular to explore if this drug can induce apoptosis in CLL B-cells from patients who are receiving therapy with ibrutinib. Using a “tartrate salt” formulation of TP-0903 we have examined CLL B-cells isolated over time from patients currently on ibrutinib for relapsed/refractory disease and in a small subset who relapsed on ibrutinib therapy for the impact of TP-0903 on CLL B-cell survival and the expression/activation status of Axl. Here we report that Axl expression remains evident in CLL B-cells from ibrutinib treated CLL patients and that TP-0903 is highly effective at inducing apoptosis in these previously treated patients even while on ibrutinib for relapsed disease.

## RESULTS

### TP-0903 is highly effective at inducing apoptosis of CLL B-cells *in vitro*

**Figure 1:**
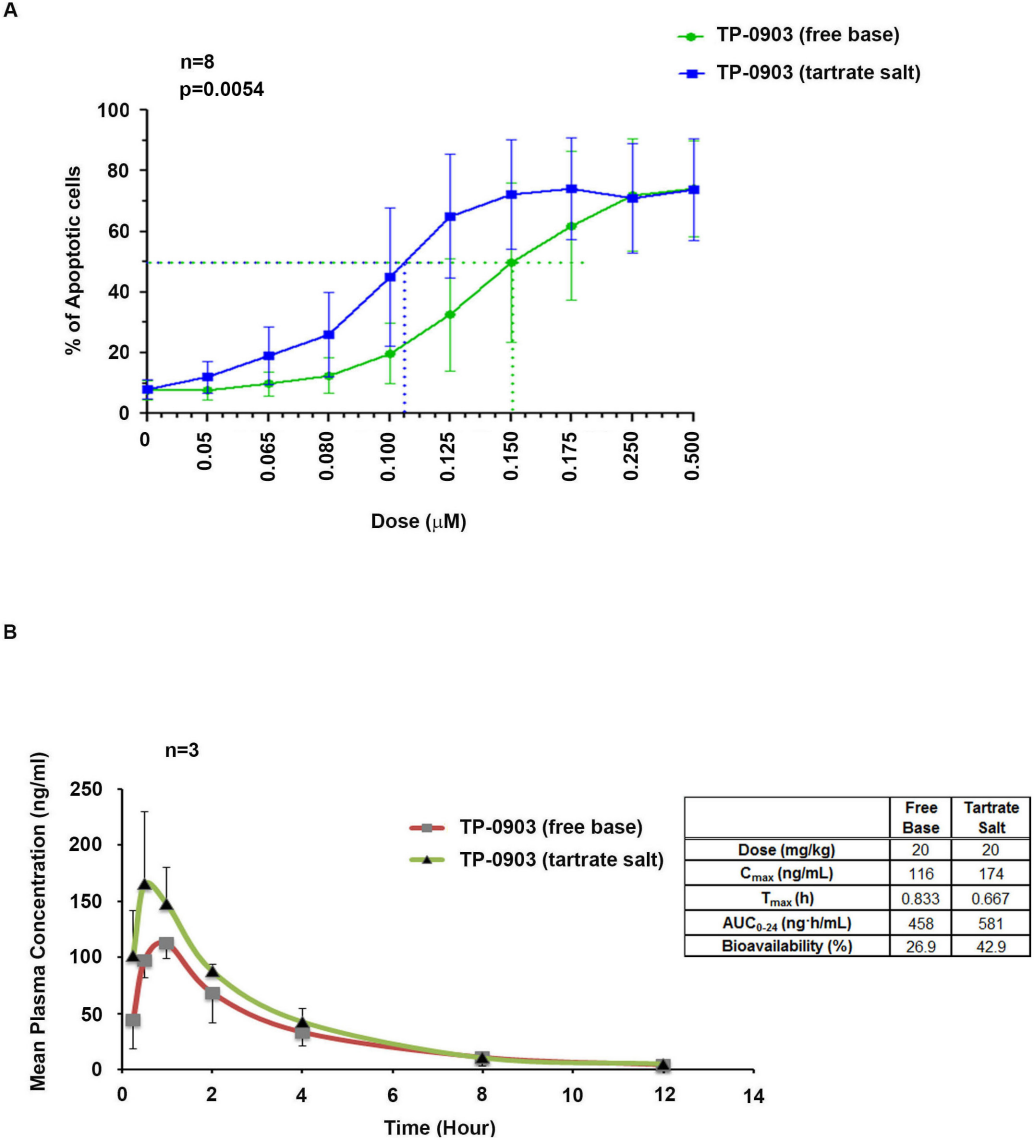
**(A)** Activity of TP-0903 (tartrate salt) vs. TP-0903 (free base) on CLL B-cell apoptosis: CLL B-cells from previously untreated patient (n=8) were treated with increasing doses (0.05-0.50 μM) of Axl inhibitor TP-0903 (tartrate salt) or TP-0903 (free base) for 24 hours. Apoptosis induction was determined and results are presented as mean values with SD. The p-value was done using a paired t-test. **(B)** Comparison of bioavailability of TP-0903 (tartrate salt) with TP-0903 (free base): The PK study was performed in Sprague-Dawley male fasted rats (n=3) after a single 20 mg/kg dose of TP-0903 (free base or tartrate salt) by oral gavage. PK parameters were calculated by Phoenix WinNonlin using a standard non-compartmental model. Details provided in a table by the figure. Bioavailability was determined for each form of TP-0903 by comparing the AUC to a control group of rats administered TP-0903 intravenously.

Earlier we have shown that administration of TP-0903 (free base) as a single agent is highly effective in inducing *in vitro* apoptosis of CLL B-cells from previously untreated patients with CLL [16]. Here we first compared the efficacy of a new TP-0903 formulation (tartrate salt) with the initial TP-0903 (free-base) in killing of CLL B-cells from previously untreated CLL patients (Figure 1A). CLL B-cells from patients were treated with increasing doses (0.05-0.50μM) of both forms of TP-0903 for 24 hours. LD_50_ values were determined from the dose response curve. We observed that the tartrate formulation remained very effective in inducing apoptosis of CLL B-cells from previously untreated CLL patients (n=8) but with a mean LD_50_ dose of 0.106 μM which is lower when directly compared to the original TP-0903 free base (mean LD_50_ 0.150) (Figure 1A). The tartrate salt formulation also has a higher peak of bioavailability than the free base formulation (Figure 1B) as determined in male Sprague Dawley rats suggesting that TP-0903-tartrate could be more useful *in vivo* than the free salt TP-0903 formulation. The tartrate salt is also superior to the free base by other pharmacokinetic parameters, including Cmax and AUC (see Figure 1B). At equal doses the free base and tartrate have equivalent toxicity profiles, so the tartrate salt allows for potentially higher drug plasma levels without additional toxicity (data not shown). This latter feature however will still need to be proven in future planned clinical trials.

### CLL B-cells from CLL patients on an ibrutinib treatment regimen express Axl and sensitive to TP-0903 (tartrate salt)

**Figure 2:**
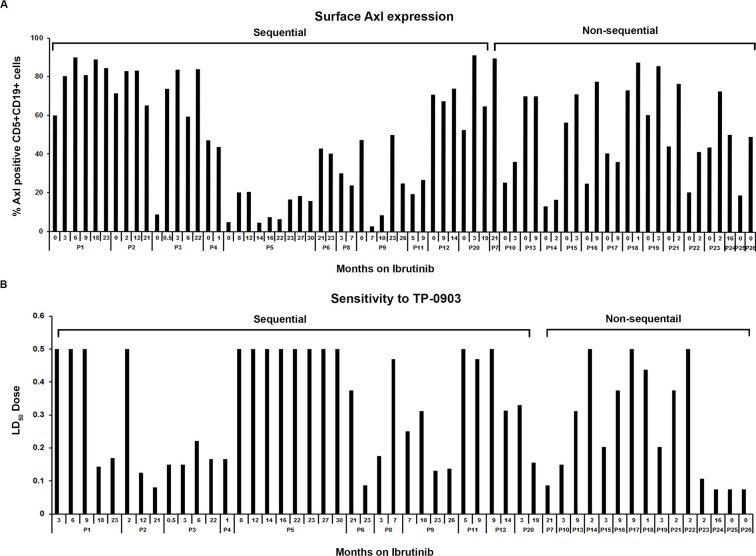
Axl expression and sensitivity to TP-0903 (tartrate salt) in CLL B-cells from patients on ibrutinib treatment **(A)** Surface Axl expression. This was determined on purified leukemic B-cells obtained from CLL patients currently on ibrutinib therapy (n=22) or who progressed while on ibrutinib (n=2; P6, P7) or who were scheduled for ibrutinib treatment (n=2; P25, P26) by flow cytometry using a specific antibody to Axl. The “0” indicates time point before ibrutinib treatment started. **(B)** Sensitivity to TP-0903. Purified CLL B-cells from blood samples of patients currently on ibrutinib therapy (n=22) or who progressed while on ibrutinib (n=2; P6, P7) or who were scheduled for ibrutinib treatment (n=2; P25, P26) were treated with increasing doses of TP-0903 (0.01–0.50μM) for 24 hours. Apoptosis induction was determined by flow cytometry after staining with Annexin/PI. Numbers depicted on horizontal axis are time points (months) when patient samples were collected from ibrutinib initiation. LD_50_ dose depicted on the vertical axis is calculated from the apoptosis measurement under the different TP-0903 concentrations. CLL patients (P1–P26) are indicated by arbitrary numbers.

Axl expression: Next we assessed the levels of surface Axl expression on CLL B-cells obtained from 26 CLL patients (7 female, 19 male) who were on ibrutinib therapy for relapsed/refractory CLL or who had progressed while on ibrutinib treatment, by flow cytometry using a specific antibody to Axl [16]. We detected Axl expression on CLL B-cells from all CLL patients tested, albeit at variable levels, with a median level of 58.9% (range 2.7-91.3%) (Figure 2A) and expression remained mostly unaltered over time during ibrutinib treatment (Figure 2A). Axl expression on CLL B-cells from sequential samples available from 11 patients (10; P1—P5, P8, P9, P11, P12, P20 were on ibrutinib therapy and 1; P6 had progressed on ibrutinib) at the initiation of ibrutinib therapy and then over two years of therapy are shown (median 43.81%; SD±30.17; range 2.7-91.3%) in Figure 2A. Sensitivity to TP-0903 treatment: To test the impact of Axl inhibition on CLL B-cell survival isolated from relapsed/refractory CLL patients (Supplementary Table 1) who were being currently treated with ibrutinib, cells were exposed *in vitro* to increasing doses of TP-0903 for 24 hours and induction of apoptosis was determined. We found 18 (69%) patients tested were sensitive to TP-0903-induced cell death (Figure 2B). Of interest, three of the four patients who had an initial LD_50_ ≥0.50μM and thus designated as insensitive to TP-0903 later were found to be sensitive to TP-0903 mediated cell death *in vitro* (Figure 2B; P1, P2, P12).

We found that TP-0903 was effective at inducing apoptosis of CLL B-cells from (Figure 2B) the ibrutinib exposed CLL patients where CLL B-cell Axl expression levels varied from 2.7-91.3%. However in one patient (P5) with low levels of Axl expression (≤20%) from samples collected at baseline and eight consecutive follow-up visits (Figure 2A), the CLL B-cells were found to be resistant to TP-0903 treatment (Figure 2B). We also found that CLL B-cells from the two patients who had clinical progression on ibrutinib (P6 and P7) were found to remain sensitive to *in vitro* TP-0903 treatment (Figure 2B). Our findings indicate that TP-0903 is active in inducing apoptosis in CLL B-cells from relapsed patients on ibrutinib therapy and is also highly effective against CLL B-cells from the two patients who progressed while on ibrutinib treatment. There were no significant differences in relation to median Axl expression on CLL B-cells who were sensitive (median 70.7%; range 2.7-91.3%) or not (median 38.8%; range 16.5-83.1%) to TP-0903 (p=0.20).

To evaluate the possible impact of TP-0903 (tartrate salt) on normal immune cells we assessed the expression of Axl, Tyro3 and MER receptors by flow cytometry on the surface of B, T and NK-cells (Supplementary Figure 1A) using peripheral blood mononuclear cells (PBMCs) isolated from normal individuals and previously untreated CLL patients. We observed significantly increased Axl expression on the surface of leukemic B-cells compared to the B-cells from normal individuals (p=0.00423) (Supplementary Figure 1B). Reduced expression of both Tyro3 and MER were detected on the surface of these leukemic B-cells compared to normal B-cells (Supplementary Figure 1B). No or minimal expression of Axl/Tyro3/MER was detected on the surface of T-cells from either healthy controls or CLL patients (Supplementary Figure 1C). Axl expression on the surface of NK-cells was very low and similar between CLL and normals. Variable Tyro3 expression and reduced MER expression were observed on the surface NK-cells from CLL PBMCs vs. normal PBMCs (Supplementary Figure 1D). Additionally, PBMCs from 6 normal individuals were cultured with increasing doses (0.01-0.50μM) of TP-0903 (tartrate salt) in 10% RPMI for 24 hours (Supplementary Figure 1E). Cells were stained with Annexin/PI and induction of apoptosis was determined by flow cytometry. Normal PBMCs did not show any significant sensitivity to TP-0903 treatment (Supplementary Figure 1E). These *in vitro* data indicate that cytotoxic effects of TP-0903 on normal immune cells should be minimal at the doses (0.01-0.50μM) that we show effectively induce apoptosis in leukemic B-cells from the ibrutinib treated cohort.

### CLL B-cells from relapsed patients express phosphorylated Axl during ibrutinib treatment

**Figure 3:**
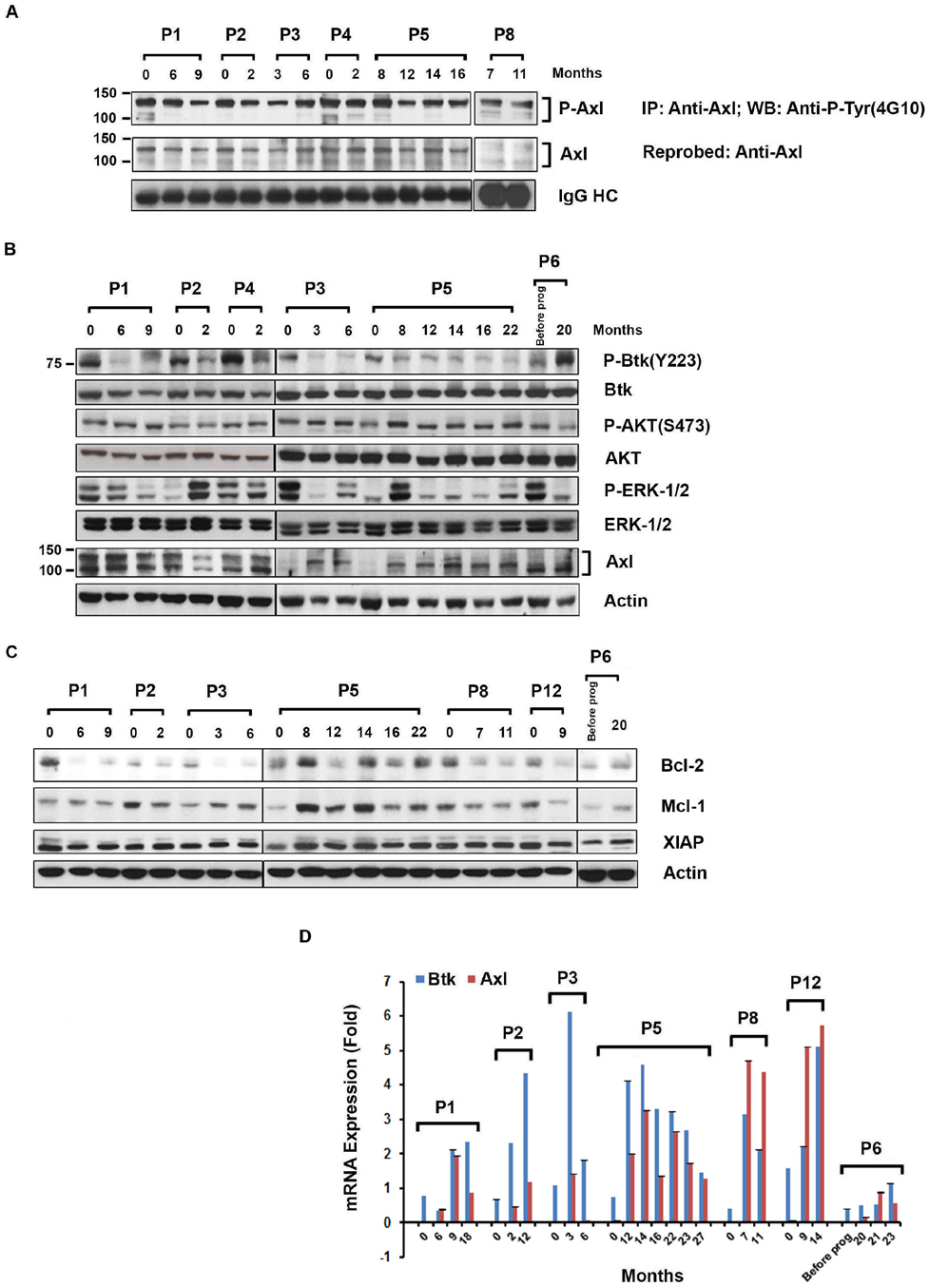
Expression of phosphorylated Axl, signal proteins and anti-apoptotic proteins in CLL B-cells from ibrutinib treated patients **(A)** Levels of P-Axl in CLL B-cells from patients exposed to ibrutinib: Axl was immunoprecipitated from the CLL B-cell lysates (n=6), followed by Western blot analysis using anti-phosphotyrosine (4G10) antibody. The blot was stripped and reprobed with an antibody to Axl. IgG HC was used as loading control. **(B)** Expression of P-Btk, P-AKT, P-ERK-1/2, and Axl in ibrutinib-exposed leukemic B-cells: Purified CLL B-cell lysates from patients currently on (n=5) or progressed (n=1; P6) while on ibrutinib were examined for the phosphorylation levels on Btk, AKT, and ERK-1/2 by Western blot analyses using specific antibodies. Blots were stripped and reprobed for total Btk, AKT and ERK-1/2. Total Axl level was also determined by Western blot using specific antibody. Actin was used as a loading control. **(C)** Expression of Bcl-2, Mcl-1 and XIAP in ibrutinib-exposed leukemic B-cells: Purified CLL B-cell lysates from patients currently on (n=6) or progressed (n=1; P6) while on ibrutinib were also examined for the expression of Bcl-2, Mcl-1 and XIAP by Western blot analyses using specific antibodies. Actin was used as a loading control. CLL patients (P1-P6, P8, P12) are indicated by arbitrary numbers. **(D)** Determination of Btk and Axl mRNA levels in CLL B-cells over time: Btk and Axl mRNA levels were measured using purified CLL B-cells from blood samples of patients currently on ibrutinib therapy (n=6) or who progressed while on ibrutinib (n=1; P6). The mRNA levels were assessed by real-time PCR using specific primers and represented as “fold change” after being normalized with the respective GAPDH mRNA levels. Data represent the average of three independent determinations with SD at each time point. CLL patients (P1–P6, P8 and P12) are indicated by arbitrary numbers. The “0” indicates time point before ibrutinib treatment started.

While CLL B-cells from previously untreated CLL patients express significantly higher levels of constitutively phosphorylated Axl compared to normal B-cells from healthy individuals [17, 18], activation status of this RTK in relapsed/refractory CLL B-cells from the ibrutinib cohort has not yet been reported. Thus, to define the Axl activation status in the latter CLL patients, we immunoprecipitated total Axl from CLL B-cell lysates of relapsed/refractory, ibrutinib exposed patients (n=6; Table 1), followed by Western blot analysis was done using an anti-phospho-tyrosine antibody to detect total P-Axl levels. We observed that Axl was constitutively phosphorylated (active) in CLL B-cells from all tested patients prior to ibrutinib therapy and this was maintained on the sequential samples of patients still on ibrutinib treatment (Figure 3A).

**Table 1:** Ibrutinib cohort with sequential visits for signaling studies

Patients	TP-0903 [LD50 (μM)]	Axl Expression (%)	Months on Ibrutinib
P1	ND	ND	0
	>0.500	90.11	6
	>0.500	80.83	9
	0.144	88.82	18
P2	ND	ND	0
	>0.500	83.06	2
	0.125	83.26	12
P3	ND	ND	0
	0.15	83.84	3
	0.2219	59.4	6
P4	ND	ND	0
	0.166	43.81	1
	ND	ND	2
P5	ND	ND	0
	>0.500	20.4	8
	>0.500	20.59	12
	>0.500	4.66	14
	>0.500	7.45	16
	>0.500	6.5	22
	>0.500	16.61	23
	>0.500	18.54	27
P8	ND	ND	0
	0.469	24.03	7
	ND	ND	11
P12	ND	ND	0
	>0.500	67.47	9
	0.313	74.18	14
P6	ND	ND	Before Progression
	ND	ND	20 (at progression)
	0.375	42.92	21 (at progression)
	0.0875	40.48	23 (at progression)

“0” indicates time point before ibrutinib treatment started.

ND: Not done (flow or drug sensitivity); used for Western blot and PCR analyses only.

**Important:** Samples were selected for study on the basis of enough available primary fresh cells.

### Status of phosphorylated Btk, AKT, ERK-1/2, Axl and anti-apoptotic proteins in CLL B-cells from patients during ibrutinib therapy

To assess the signal pathway status of CLL B-cells from patients undergoing ibrutinib therapy particularly in relation to the activity of TP-0903, we obtained CLL B-cell lysates from the ibrutinib exposed cohort (n=5) and the ibrutinib progressed patients (n=1) at sequential time points (Table 1). These lysates were further analyzed for phosphorylation status of Btk, AKT and ERK-1/2 by Western blot analyses. As expected, phosphorylation on Btk (Y223) remained significantly reduced in CLL B-cells during the ibrutinib treatment compared to their respective baseline samples while the total Btk protein levels remained relatively unchanged (Figure 3B). Also as expected CLL B-cells from the CLL patient (P6), who progressed while on ibrutinib showed an increase in phospho-Btk (Y223) level (Figure 3B). It has been established that Axl mediates its anti-apoptotic effects via AKT activation [19]. Importantly, CLL B-cells from all of these patients also expressed constitutively high levels of phospho-AKT (Ser473) before and during the ibrutinib treatment (Figure 3B) indicating continued AKT activation. However, levels of P-ERK-1/2 in CLL B-cells mostly remain reduced in ibrutinib exposed patients over time compared to their respective baseline (pre-ibrutinib) samples (Figure 3B). In addition, CLL B-cells from six sequentially studied patients were analyzed for total Axl expression before and during the ibrutinib treatment by Western blot analysis. While on ibrutinib, CLL B-cells from all six CLL patients continued to express Axl at variable levels or increased their Axl expression (n=2; P3, P5) levels during the follow-up period (Figure 3B). Western blot analyses showed that *in vivo* ibrutinib treatment decreased Bcl-2 expressions in CLL B-cells from most of the patients (n=7) tested and Mcl-1 expressions in P2, P8 and P12 but had no effect on XIAP expressions in these patients (Figure 3C). Altogether, we found constitutively active Axl, its downstream P-AKT, and unaltered expressions of both anti-apoptotic proteins Mcl-1 and XIAP in CLL B-cells from ibrutinib treated patients. This data prompted us to explore the effectiveness of Axl inhibitor TP-0903 in killing of CLL B-cells from this cohort.

### Btk and Axl mRNA levels in CLL B-cells from ibrutinib exposed cohort

It has been reported that BTK mRNA level in cells from CLL patients are significantly higher than that observed in normal B-cells [20]. To see the effect of ibrutinib on the Btk mRNA expression in this study cohort, we examined the status of Btk mRNA levels over time in CLL B-cells from the ibrutinib exposed CLL cohort (n=7). Additionally since we observed continued Axl expression at the protein level in the ibrutinib treated cohort we wanted to also evaluate Axl mRNA levels in CLL B-cells from these patients. Real-time PCR was done using cDNA from purified CLL B-cells from the six patients placed on ibrutinib and one ibrutinib-relapsed patient. Baseline mRNA levels were compared to the respective sequential samples collected from the CLL patients. CLL B-cells from all patients continued to express increased levels of both Axl and Btk mRNA over time (Figure 3D). Thus in CLL B-cells for patients P1, P2, P6, and P12, there were detectable levels at baseline and then increasing levels of Btk mRNA over time while P3, P5, and P8 had detectable baseline Btk mRNA levels which after an initial increase dropped gradually over time but still were detectable at the last study time point. On the other hand, Axl mRNA levels in CLL B-cells increased over time in 3 patients (P2, P8 and P12), and variable but high levels of the RTK mRNA were detected in the other 4 patients (P1, P3, P5, and P6).

### TP-0903 (tartrate salt) inhibits total tyrosine-phosphorylation on Axl in CLL B-cells from patients exposed to ibrutinib

**Figure 4:**
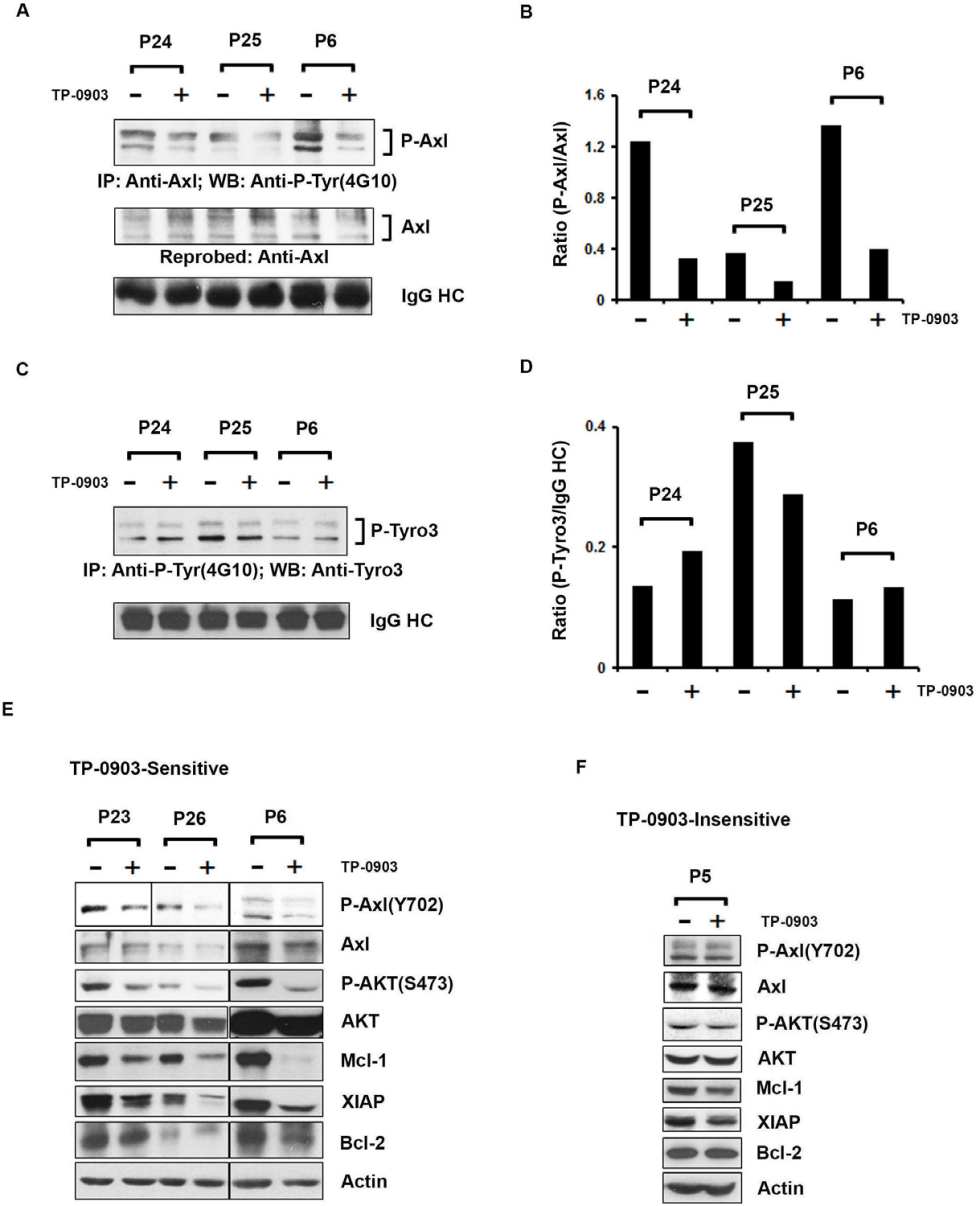
TP-0903 (tartrate salt) modulates CLL B-cell signaling proteins **(A)** TP-0903 inhibits tyrosine phosphorylation on Axl in CLL B-cells from ibrutinib treated cohort:Axl was immunoprecipitated from the DMSO or TP-0903 treated (20–24 hours) CLL B-cell lysates (n=3), followed by Western blot analysis using a global anti-phosphotyrosine (4G10) antibody. The blot was stripped and reprobed with an antibody to Axl. IgG HC was used as loading control. **(B)** Levels of P-Axl expression in CLL B-cells are quantified using ImageJ software and are represented as a ratio of P-Axl/Axl in the bar graph. **(C)** Effect of TP-0903 on Tyro3 phosphorylation: Total tyrosine phosphorylated proteins were immunoprecipitated from the DMSO or TP-0903 treated CLL B-cell lysates (n=3) used above using anti-phosphotyrosine antibody (4G10), followed by Western blot analysis using anti-Tyro3 antibody. IgG HC was used as loading control. **(D)** Levels of P-Tyro3 expression in CLL B-cells are quantified using ImageJ software and are represented as a ratio of P-Tyro3/IgG HC in the bar graph. **(E)** Impact of TP-0903 on Axl phosphorylation at kinase domain (Y702) and downstream signaling: DMSO or TP-0903–treated CLL B-cell lysates from TP-0903-sensitive patients (n=3) were analyzed for the status of phosphorylation at Axl (Y702) and AKT (S473) by Western blot analyses using specific antibodies. Respective blots were then stripped and reprobed with Axl or AKT antibody. TP-09003 treated same lysates were also used to determine the expression of anti-apoptotic proteins Mcl-1, XIAP, and Bcl-2 in Western blot using specific antibodies. Actin was used as a loading control. CLL patients (P23, P24; on ibrutinib treatment, P25, P26; scheduled for ibrutinib treatment and P6; progressed while on ibrutinib treatment) are indicated by arbitrary numbers. **(F)** Impact of TP-0903 on P-Axl (Y702), P-AKT (S473) and downstream signaling in CLL B-cells from a TP-0903-insensitive patient:DMSO or TP-0903 (0.50 μM)–treated CLL B-cell lysates from patient ‘P5’ were analyzed for the status of phosphorylation at Axl (Y702) and AKT (S473) by Western blot analyses using specific antibodies. Respective blots were then stripped and reprobed with Axl or AKT antibody. TP-09003 treated same lysates were also used to determine the expression of anti-apoptotic proteins Mcl-1, XIAP, and Bcl-2 by Western blot using specific antibodies. Actin was used as a loading control. CLL patient (P5) is indicated by arbitrary number.

We have previously shown that TP-0903 reduces Axl phosphorylation in CLL B-cells [16]. To further validate that TP-0903 is able to target and reduce phosphorylation of Axl in CLL B-cells from ibrutinib exposed patients, purified CLL B-cells from the ibrutinib treated cohort (n=3; Table 2) were treated with a sub-lethal dose of TP-0903 (dose determined from the TP-0903 dose-response curve) (Table 2) or left untreated for 16-20 hours. Axl was then immunoprecipitated from the cell lysates, followed by Western blot analysis using a phospho-tyrosine specific antibody (4G10) to detect P-Axl levels. We observed that TP-0903 reduced total tyrosine-phosphorylation levels of Axl (Figure 4A and 4B), as expected. Recently we detected that CLL B-cells co-express phosphorylated Axl and Tyro3 [16, 17]. To further examine if TP-0903 was also able to target P-Tyro3, tyrosine-phosphorylated proteins were pulled down from TP-0903-treated CLL B-cell lysates by IP using 4G10-antibody, followed by Western blot analysis to detect Tyro3. We found that TP-0903 was unable to target P-Tyro3 (Figure 4C and 4D) in CLL B-cells from ibrutinib treated patients at the doses known to target P-Axl. This is consistent with our earlier report [16] on the target specificity of TP-0903 towards Axl in CLL B-cells.

**Table 2:** TP-0903 treated cohort for signal protein studies

Patients	TP-0903 [LD50 (μM)]	Sub-lethal dose used (μM)	Axl Expression (%)	On Ibrutinib (Months)
P5	>0.500	0.5	16.61	23
P6	0.375	0.25	42.92	21 (at progression)
P23	0.106	0.08	72.72	2
P24	0.075	0.06	50.25	16
P25^*^	0.075	0.075	18.88	0
P26^*^	0.075	0.07	49.13	0

* Patients included as baseline of ibrutinib study and “0” indicates time point before ibrutinib treatment started.

Treatment dose (sub-lethal) was determined from the individual patient’s TP-0903 dose response curve.

### TP-0903 (tartrate salt) inhibits Axl downstream P-AKT (S473) and expression of anti-apoptotic proteins in ibrutinib-exposed CLL B-cells

Tyrosine residues at positions 702 and 703 of Axl have been shown to be phosphorylated upon GAS6 binding and the consequent activation of the molecule. Therefore, to further examine if TP-0903 could target tyrosine phosphorylation at the kinase domain of Axl [21], TP-0903 exposed CLL B-cell lysates were analyzed to detect the status of P-Axl (Y702) by Western blot using a phospho-specific antibody. Indeed, TP-0903 treatment efficiently reduced phosphorylation at the Y702 residue in the Axl kinase domain in CLL B-cells from TP-0903-sensitive patients (n=3; Figure 4E and Supplementary Figure 2A). We also examined the impact of TP-0903 on the Axl downstream signaling molecule, AKT, and levels of anti-apoptotic proteins, Mcl-1, Bcl-2 and XIAP in CLL B-cells by Western blot analyses using specific antibodies. TP-0903 was indeed able to inhibit AKT phosphorylation and reduced expressions of both Mcl-1 and XIAP in CLL B-cells from ibrutinib exposed patients but had no clear effect on Bcl-2 expression in sensitive patients (Figure 4E and Supplementary Figure 2B-2E). However, TP-0903 treatment did not affect the phosphorylation levels on Axl(Y702) or AKT(S473) in CLL B-cells from a TP-0903-insensitive patient P5 (Figure 4F and Supplementary Figure 3A, 3B). The 0.50 μM dose of TP-0903 also had insignificant effect on both Mcl-1 and XIAP expression in CLL B-cells from patient P5 and had no effect on the Bcl-2 level (Figure 4F and Supplementary Figure 3C-3E).

### Prognostic factors and sensitivity of CLL B-cells to TP-0903 (tartrate salt) treatment

Finally, we analyzed whether established biologic prognostic factors had any association with the sensitivity of CLL B-cells to TP-0903 treatment in this CLL cohort (n=26). There were no significant differences in FISH, *IgVH* mutation status, CD49d, or Rai stage between the sensitive and insensitive LD_50_ CLL groups. The median number of treatments prior to initiation of ibrutinib did not differ significantly between sensitive (2.0; range 0-8) and insensitive (2.5; range 0-12) groups (p=0.47). There was a significant association for CD38^+^ CLL B-cells to have higher apoptosis induction levels when exposed to TP-0903 (p=0.02) [Supplementary Figure 4A] in patients exposed to ibrutinib. There was also a trend for ZAP70^+^ CLL B-cells to be more sensitive to TP-0903 treatment (Supplementary Figure 4B). Sensitive subjects also tended to be younger with a median age of 64 (n=18) vs. 73 (n=8) years (p=0.08).

## DISCUSSION

Patients with CLL whether previously untreated or relapsed will achieve a long period of progression-free survival on ibrutinib, but ibrutinib is not curative and resistance develops. Because of these important clinical issues our goal here was primarily to assess if another cell target could be effective in the induction of CLL leukemic B-cell apoptosis. To do this we focused on the activity of TP-0903, a highly specific Axl RTK inhibitor in ibrutinib treated CLL patients. Our findings indicate that Axl inhibitor, TP-0903 was active at inducing *in vitro* apoptosis in CLL B-cells from relapsed patients placed on ibrutinib therapy and also was highly effective against CLL B-cells from two patients who had progressed while on ibrutinib treatment. We found that sensitivity of the leukemic B-cells to TP-0903 induced apoptosis remained consistent over time in the patients where we were able to obtain sequential blood samples. The drug we tested is orally bioavailable and is now being used in a first human clinical trial in patients with advanced solid tumors (http://clinicaltrials.gov identifier NCT02729298).

In our study, Axl RTK expression remained evident in all tested patients from the initial start of ibrutinib therapy and then over a two year follow-up period. Here we wanted to point out that relapsed/refractory CLL patients still actively express both Axl mRNAs and protein in spite of being on long term ibrutinib therapy. Since the role of Axl is established for drug resistance in many cancers and there is continued Axl expression in CLL B-cells from patients on ibrutinib therapy encourages us to consider Axl as a potential therapeutic target in this CLL cohort.

In this study we also observed the efficacy of TP-0903 apoptosis induction in a high risk group of relapsed/refractory CLL patients who received a median number of two prior therapies. In addition, we found that seven of eight 17p- and all seven 11q- patients were sensitive to *in vitro* apoptosis induction by Axl inhibitor TP-0903. In support of our work induction of leukemic B-cell apoptosis was reported recently by TP-0903 treatment in CLL patients before and after oral ibrutinib therapy [22]. When we assessed TP-0903 apoptosis induction for CLL B-cells association with novel prognostic factors, we found that with a higher expression of CD38 was positively associated with higher levels of apoptosis. Additionally, the impact of TP-0903 on normal immune cells was found to be very low based on apoptosis induction and is consistent with the low expression of Axl on both T and NK-cells.

All CLL patients who had been treated with ibrutinib expressed Axl at variable but detectable levels. We also noted the presence of phosphorylated Axl in CLL B-cells obtained from all tested patients. Axl mRNA levels were found to be present in CLL B-cells from all seven studied sequential patients and increased in six patients on ibrutinib, indicating that active transcription of this RTK continued while on ibrutinib treatment. Despite being on BTK signal inhibitor therapy, Axl was both actively transcribed and translated as evident from the presence of the RTK on CLL B-cell plasma membrane. Thus Axl continues to be expressed in ibrutinib treated CLL patients and is likely an attractive target in these leukemic B-cells using TP-0903. In addition we found that TP-0903 targeted total tyrosine phosphorylation on Axl and this inhibition included the activating phosphorylation site at the Y702 residue [21] in CLL B-cells from patients on ibrutinib treatment. If TP-0903 were to be added to CLL patients still on ibrutinib therapy this may be of additional clinical benefit in that we showed earlier that combined treatment of TP-0903 with ibrutinib or a reversible BTK inhibitor, TP-4216 (Tolero Pharmaceuticals) of CLL B-cells from previously untreated CLL patients augmented leukemic B-cell apoptosis than with each agent alone [16].

Limitations of this study include the relatively small sample size; however, we were able to repeatedly test the same patients over time for both Axl expression and sensitivity to TP-0903 with consistent results. Here we also determined if CLL B-cells were continuing to express BTK mRNA and indeed we found an increased transcription of Btk in some patients despite reduced phosphorylated Btk protein levels.

While we suspect that Axl inhibition plays a key role in the CLL B-cell apoptosis we cannot rule out off target effects of TP-0903 in the induction of apoptosis of CLL B-cells as we found high levels of apoptosis even in CLL B-cells with low levels of Axl. While TP-0903 is highly specific for Axl [23] there are other kinases that can potentially be modulated by TP-0903 [24]. We did rule out that the apoptosis induction was related to Tyro3 on CLL B-cells since we did not find any inhibitory effect of TP-0903 on phosphorylated Tyro3 in CLL B-cells. We suspect that the effectiveness of any drug treatment will also depend on other features including prior therapy exposure history and the biology of the cell where the presence of more adverse prognostic risk factors like can render the cell more resistant. However, we are encouraged to believe that the primary impact of TP-0903 is related to its targeting of Axl RTK as exposure of CLL B-cells to TP-0903 did consistently result in reduction of phosphorylated Axl and also reduced phosphorylated AKT and levels of anti-apoptotic proteins, Mcl-1 and XIAP. These latter findings help to explain the *in vitro* capacity of the drug to induce apoptosis since both AKT activation status and levels of the anti-apoptotic proteins Mcl-1 and XIAP are known to play key roles in apoptosis status of CLL B-cells [25, 26]. While not the focus of this paper, which was primarily to show the high degree of CLL B-cell *in vitro* apoptosis found with nanomolar doses of TP-0903, we continue to interrogate which biologic features of the CLL B-cell render them sensitive to this drug.

In summary, we have found that the orally bioavailable drug TP-0903 is capable of dramatic apoptosis induction of CLL B-cells from relapsed/refractory patients being treated with ibrutinib. The TP-0903 formulation we have tested is known to achieve high peak serum concentrations and is now being tested in initial phase 1 trials in humans. The *in vitro* data we have obtained supports the further testing of this drug when used alone in subsequent phase 1 and 2 trials in hematologic malignancies including CLL.

## MATERIALS AND METHODS

### Patient cohort demographics, B-cell or PBMC isolation and cell culture

The study cohort included 26 relapsed/refractory CLL patients. Details are available in the supplementary materials and methods. Previously untreated patients (n=25) also were included in the study as needed.

### Reagents

Detailed information of the reagents used is provided in the supplementary materials and methods.

### Treatment of PBMCs or CLL B-cells with TP-0903 and induction of apoptosis

Our prior work established that the mean LD_50_ dose of TP-0903 was at 0.14 μM [16] for previously untreated CLL patients. We also found that more than 90% of CLL B-cells from previously untreated CLL patients were killed at ≤ 0.50μM (unpublished data). A more bioavailable formulation of TP-0903 (tartrate salt) was made available to us by Tolero pharmaceuticals. LD_50_ values for untreated CLL patients (n=25) with TP-0903 (tartrate salt) is 0.10 μM ± 0.04 versus 0.32 μM ± 0.18 [mean ± standard deviation (SD)] for the cohort of ibrutinib treated CLL patients (n=26) reported here (Supplementary Figure 5). Thus with this data we decided to designate any dose response where we found LD_50_ ≤ 0.50 μM for the CLL B-cells from the studied cohort as exhibiting sensitivity to the drug. Detailed procedure is provided in the supplementary.

### Determination of Axl or Tyro3 or MER expression by flow cytometry

We determined the relative expression level of Axl or Tyro3 or MER on B or T or NK-cells from healthy controls and previously untreated patients by flow cytometry using a specific antibody to Axl [17, 27] or Tyro3 or MER. For the detection of B/T/NK-cells, chromogen-conjugated antibody to CD19 or CD5 or CD3 (BD Bioscience) was used to stain the cells prior to analysis on the flow cytometer (BD Calibur). The *gating strategy for Axl positivity on CD5+CD19+ B-cells is shown in Supplementary Figure 6. Positive expression of* Axl, Tyro3 or MER was determined by comparing with respective isotype control.

### Immunoprecipitation and western blot analysis

Purified CLL B-cells (4.0 × 10^6^/ml) treated with DMSO or sub-lethal doses of TP-0903 for 16-20 hours were lysed in NP40-lysis buffer, and whole cell extract was prepared as described previously [17, 27]. Detailed procedures are also described in the supplementary section.

### Reverse transcription polymerase chain reaction (PCR)

Comparative real-time PCR was performed in triplicate. Relative expression was calculated using the comparative Ct method [28] and presented here as “fold expression”. Detailed procedure is available in the supplementary section.

### Pharmacokinetics (PK) of TP-0903 free base vs. TP-0903 tartrate salt

The pharmacokinetics study was conducted by Tolero Pharmaceuticals. See supplementary materials and methods for a detailed description of this work.

### Statistical analysis

Statistical analysis is discussed in the supplementary text.

## SUPPLEMENTARY MATERIALS FIGURES AND TABLE




