# Supplementary material for: Chronic lymphocytic leukemia cells from ibrutinib treated patients are sensitive to Axl receptor tyrosine kinase inhibitor therapy
Source: Oncotarget. 2018 Dec 14;9(98):37173–84. doi: 10.18632/oncotarget.26444 (PMC6324680; doi:10.18632/oncotarget.26444)
Supplement: Supplementary file 1 [file oncotarget-09-37173-s001.pdf]

Chronic lymphocytic leukemia cells from ibrutinib treated patients are sensitive to Axl receptor tyrosine kinase inhibitor therapy

SUPPLEMENTARY MATERIALS

Patient cohort demographics, B-cell or peripheral blood mononuclear cell (PBMC) isolation and cell culture

The patient cohort (n=26) included in the study were considered very high risk for disease progression based on FISH panel assessment (i.e. eight were 17p- and seven were 11q- by FISH) and *IgVH* mutation status (17 unmutated). Twenty two (n=22) patients were treated with ibrutinib for progressive disease, two (P6 and P7) had relapsed while on ibrutinib. Two patients (P25 and P26; one relapsed after chemo-immunotherapy and the other relapsed while on rituximab) were scheduled for ibrutinib treatment and thus considered as baseline patients (pre ibrutinib therapy) for the study. Out of 26 patients, 11 were studied sequentially from the initial start of ibrutinib therapy and then over a two year follow-up period. The median number of prior therapies was 2.0, range (0-12). Patients provided initial blood samples at a median of 2.9 months (range: 3 – 21.5 months) after ibrutinib therapy initiation for the *in vitro* studies reported here. All patients provided written informed consent according to the Declaration of Helsinki to the Mayo Clinic Institutional Review Board, which approved these studies.

Primary CLL B-cells were isolated and purified from blood of the CLL patients using the RosetteSep B-cell enrichment kit (Stem Cell Technologies). The purification range of $CD5^+/CD19^+$ CLL B-cells was >95-99%. Cells were cultured in serum-free AIM-V (GIBCO) medium as needed. PBMCs were isolated from blood by Ficoll.

Reagents

A high-affinity orally bioavailable Axl inhibitor TP-0903 (tartrate salt) and TP-0903 (free base) was provided by Tolero Pharmaceuticals Inc., Bcl-2 antibody was purchased from BD Pharmingen and antibodies to Actin and Axl were purchased from Santa Cruz Biotechnologies. Tyro3 and MER antibodies were obtained from Abcam and Lifespan Biosciences, respectively. Phosphotyrosine mouse monoclonal antibody (4G10) was obtained from Millipore. All other antibodies were obtained from Cell Signaling Technology.

Treatment of CLL B-cells with TP-0903 and induction of apoptosis

CLL B-cells (2 x 10^6 cells/ml) from CLL patients (previously untreated or ibrutinib exposed) were treated with increasing doses of TP-0903 (either freebase or tartrate salt) (0.01–0.50μM) for 24 hours in serum-free AIM-V media. PBMCs (2 x 10^6 cells/ml) from healthy normals were also treated with increasing doses of TP-0903 (tartrate salt) (0.01–0.50μM) for 24 hours in 10% RPMI medium. TP-0903 treated PBMCs or CLL B-cells were harvested, and induction of apoptosis determined by flow cytometry (FACScan, Becton Dickinson) after staining the cells with Annexin V/propidium iodide (PI) as described [16].

Immunoprecipitation and western blot analysis

For immunoprecipitation (IP) experiments, 0.2–0.3 mg of CLL B-cell lysates was used to IP using appropriate antibodies as described elsewhere [16]. The precipitated immune complex was electrophoresed on SDS-polyacrylamide gels and transferred onto nitrocellulose membranes, and proteins of interest were detected using specific antibodies. CLL B-cell lysates were analyzed for the expressions of Axl, XIAP, Bcl-2, and Mcl-1by Western blot. CLL B-cell lysates were also analyzed for the phosphorylation status of Axl, Btk, AKT and ERK-1/2 using specific antibodies by Western blot.

Reverse transcription polymerase chain reaction (PCR)

Total cellular RNA was extracted using Purelink Mini Kit (Invitrogen) and 1 μg was reverse transcribed using the SuperScript® III First Strand Synthesis Kit (Invitrogen) and PCR was performed using the SYBR Green PCR core Master Mix (Applied Biosystems, Foster City, CA, USA) on a 7500 Real-Time PCR System (Applied Biosystems) according to the manufacturer's instructions. GAPDH was used for cDNA normalization. Btk, Axl and GAPDH primers were purchased from SABiosciences (Frederick, MD, USA).

Pharmacokinetics (PK) Study

The study was done using fasting, male Sprague Dawley rats. Both the free base form of TP-0903 and the tartrate salt were formulated in 20% solutol. The free base of TP-0903 was formulated at 5.0 mg/ml (PO) and dosed by oral gavage (18.2 mg/kg). The tartrate salt of TP-0903 was formulated at 6.5 mg/ml to account for the added weight of the salt and dosed by oral gavage (14.5 mg/kg). Plasma samples were taken at 0.25, 0.5, 1, 2, 4, 8, 12 and 24 h and analyzed for TP-0903 concentration by LC-MS/MS with reference to a previously determined standard curve. Pharmacokinetic parameters were calculated using a non-compartmental approach with Phoenix WinNonlin 6.3 (Pharsight, Mountain View, CA, USA).

Statistical analysis

CLL prognostic factors (i.e. ZAP70, FISH, *IgVH* mutation status, Rai stage, CD38, and CD49d) were evaluated for association with TP-0903 apoptosis induction data of the respective CLL B-cells. Associations between prognostic factors and TP-0903 drug sensitivity were computed using the Kruskal-Wallis test for continuous variables and Chi-square test for categorical variables. SAS 9.4 (SAS Institute, Cary, NC, USA) was used for data analysis. The p-values were generated using a paired t-test.

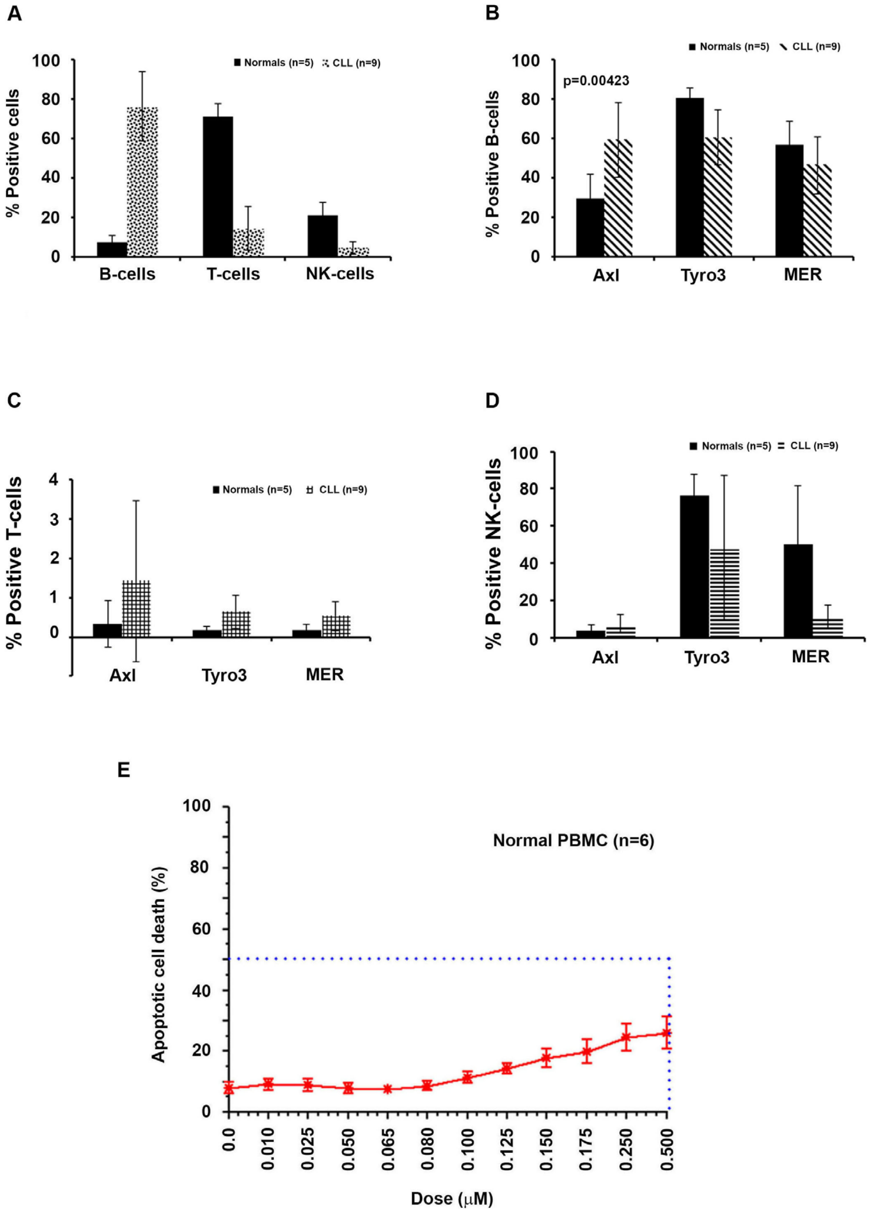

Supplementary Figure 1: Expression of Axl, Tyro3 and MER on B, T and NK-cells from healthy control vs. CLL-patients and the impact of TP-0903 (tartrate salt) on normal immune cells. **(A)** Expression levels of $CD5^{+}CD19^{+}$ leukemic B, $CD3^{+}$ T and $CD19^{-}/CD3^{-}$ NK-cells in CLL patients (n=9) vs. normal individuals (n=5). Surface Axl or Tyro3 or MER expression was determined on the B-cells **(B)** or T-cells **(C)** or NK-cells **(D)** using PBMCs either from normal controls (n=5) or untreated CLL patients (n=9) by flow cytometry using a specific antibody to Axl/Tyro3/MER. **(E)** PBMCs isolated from normal, healthy individuals (n=6) were treated with increasing doses of TP-0903 (0.01–0.50 μM) and cultured in 10% RPMI medium for 24 hours. Apoptosis induction was determined and results are presented as mean values with SD. The p-value was done using a paired t-test.

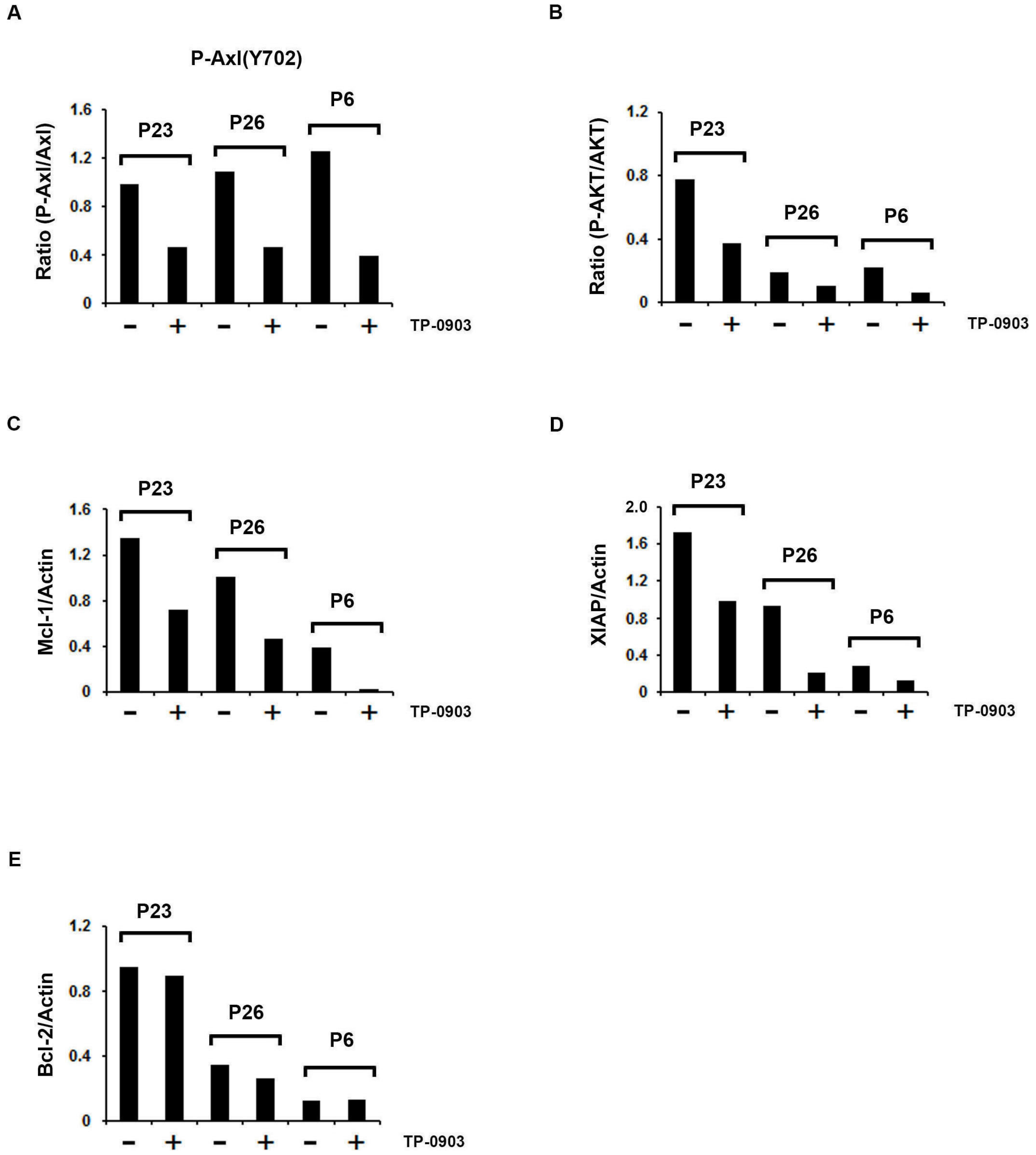

Supplementary Figure 2: Effect of TP-0903 (tartrate salt) on P-Axl(Y702) and downstream signaling in CLL B-cells from TP-0903 (tartrate salt) sensitive patients (n=3). All quantification of proteins shown in this figure were done by using ImageJ software: **(A)** Levels of P-Axl(Y702) expression in CLL B-cells were evaluated by Western blot and were then quantified and represented as a ratio of P-Axl/Axl in the bar graph. **(B)** Levels of P-AKT (S473) expression in CLL B-cells were evaluated by Western blot and were then quantified and represented as a ratio of P-AKT/AKT in the bar graph. **(C)** Mcl-1 expression levels in CLL B-cells were evaluated by Western blot and then were quantified and represented as a ratio of Mcl-1/Actin in the bar graph. **(D)** XIAP expression in CLL B-cells was evaluated by Western blot and then was quantified and represented as a ratio of XIAP/Actin in the bar graph. **(E)** Bcl-2 expression in CLL B-cells was evaluated by Western blot and then was quantified and represented as a ratio of Bcl-2/Actin in the bar graph. CLL patients (P23; on ibrutinib therapy, P26; just before ibrutinib therapy and P6; progressed while on ibrutinib therapy) are indicated by arbitrary numbers.

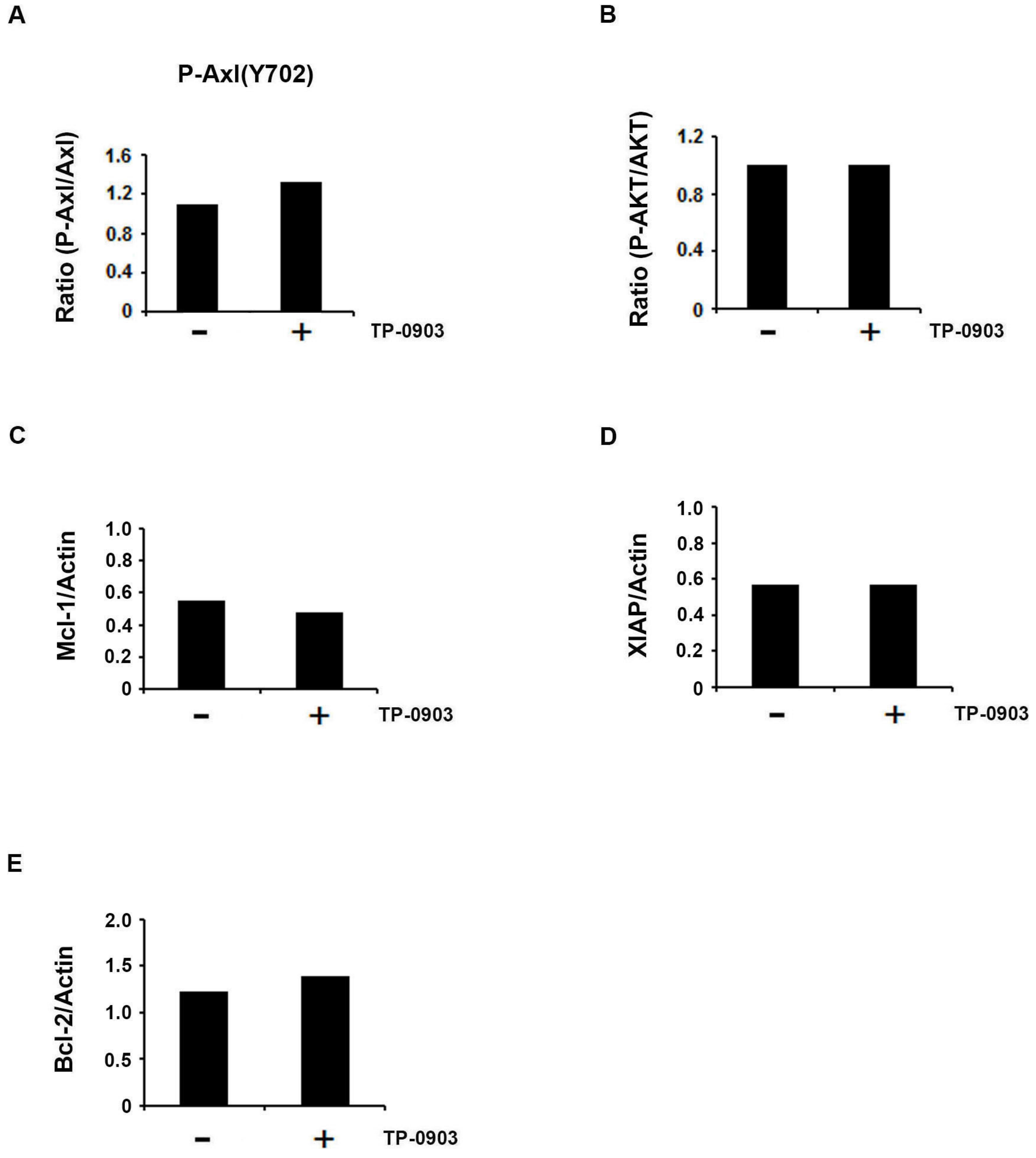

Supplementary Figure 3: Effect of TP-0903 (tartrate salt) on P-Axl(Y702) and downstream signaling in CLL B-cells from TP-0903 (tartrate salt) insensitive patient on ibrutinib. (A) Levels of P-Axl (Y702) expression in CLL B-cells from a CLL patient resistant to TP-0903 were evaluated by Western blot and then were quantified and represented as a ratio of P-Axl/Axl in the bar graph. **(B)** Levels of P-AKT (S473) expression in CLL B-cells from a representative CLL patient resistant to TP-0903 were evaluated by Western blot and then were quantified and represented as a ratio of P-AKT/AKT in the bar graph. **(C)** Mcl-1 expression levels in CLL B-cells were evaluated by Western blot and then were quantified and represented as a ratio of Mcl-1/Actin in the bar graph. **(D)** XIAP expression in CLL B-cells was evaluated by Western blot and then was quantified and represented as a ratio of XIAP/Actin in the bar graph. **(E)** Bcl-2 expression in CLL B-cells was evaluated by Western blot and then was quantified and represented as a ratio of Bcl-2/Actin in the bar graph. CLL patient P5 is indicated by arbitrary number.

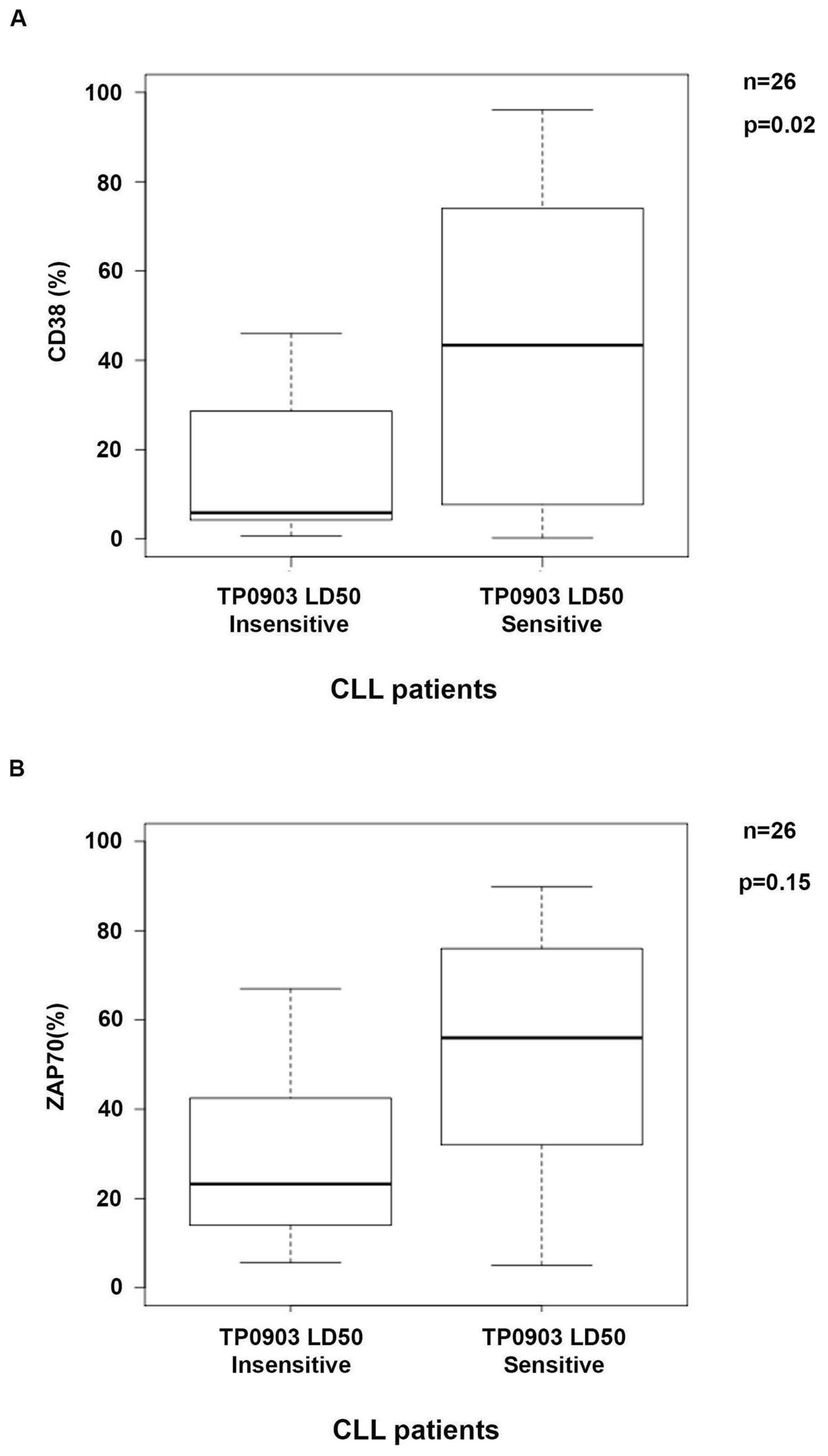

Supplementary Figure 4: Association of CLL patients (n=26) with a positive CD38 and ZAP70 status to TP-0903 (tartrate salt) treatment. **(A)** Comparison of TP-0903 LD_{50} sensitivity in cells from CLL patients to the percent CD38 expression levels (p=0.02). **(B)** Comparison of TP-0903 LD_{50} sensitivity in cells from CLL patients with percent ZAP70 expression levels (p=0.15).

A

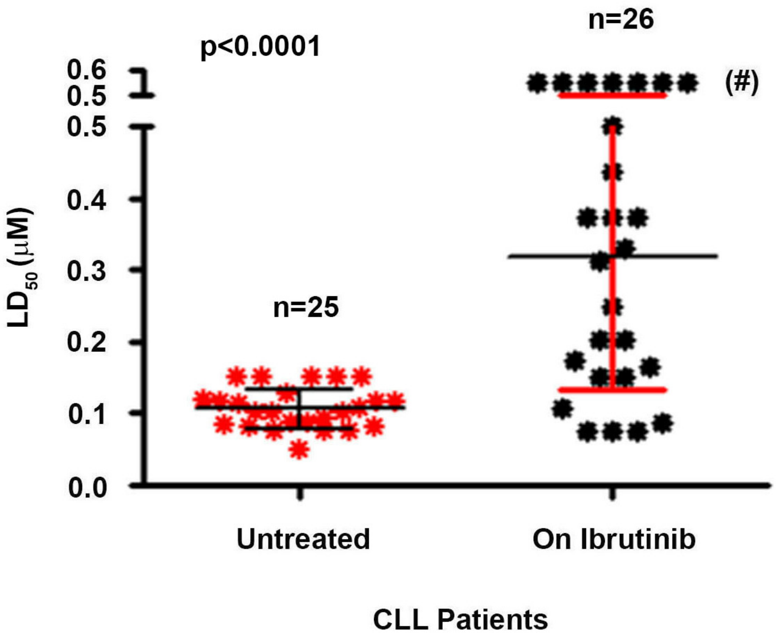

Supplementary Figure 5: Sensitivity of a cohort of untreated CLL patients vs. a cohort of CLL patients treated with ibrutinib to TP-0903 (tartrate salt). CLL B-cells from previously untreated patients (n=25) or patients on ibrutinib (n=26) were treated with increasing doses (0.01-0.50 μM) of TP-0903 for 24 hours. Numbers depicted on the vertical axis are LD_{50} values of TP-0903 for these CLL cohorts and were determined from the dose response curve. # Indicates $LD_{50} > 0.50$ μM. The horizontal lines represent the mean value and the ± one standard deviation. The p-value was done using a paired t-test.

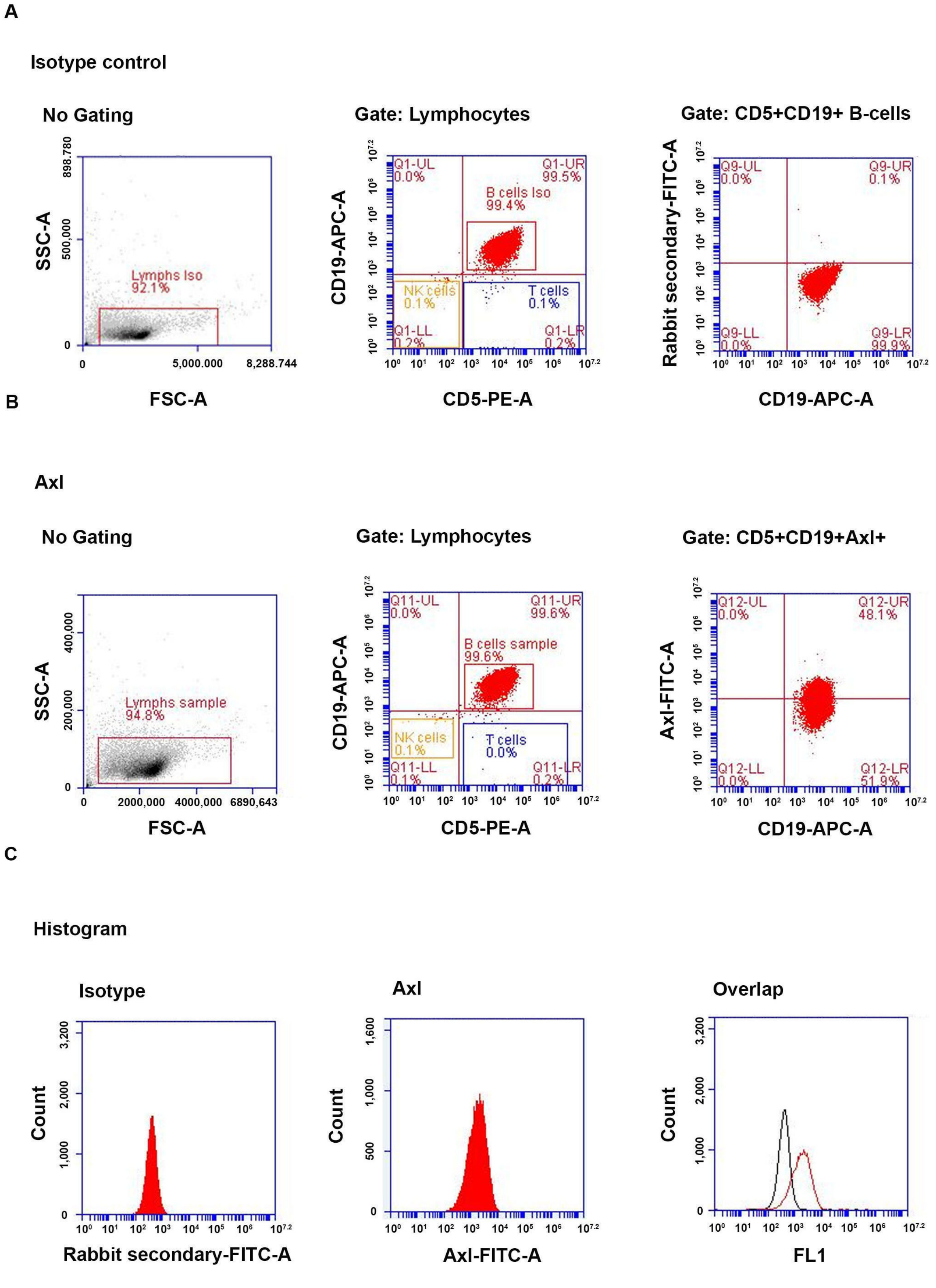

Supplementary Figure 6: Representative flow plots for Axl staining. **(A)** CD5 and CD19 positive B-cells gating for isotype control. **(B)** CD5 and CD19 positive B-cells gating for Axl staining. **(C)** Representative histogram for Axl staining.

Supplementary Table 1: Characteristics of Ibrutinib cohort

See Supplementary File 1
