# Supplementary material for: Chronic lymphocytic leukemia cells from ibrutinib treated patients are sensitive to Axl receptor tyrosine kinase inhibitor therapy
Source: Oncotarget. 2018 Dec 14;9(98):37173–84. doi: 10.18632/oncotarget.26444 (PMC6324680; doi:10.18632/oncotarget.26444)
Supplement: Supplementary file 2 [file oncotarget-09-37173-s002.docx]

Supplementary Table 1: Characteristics of Ibrutinib cohort

|  | **On Ibrutinib** |  |  |  | **CD38** | **CD49d** | **RAI** | **ZAP70** | **TP0903** |  | **% Death ^Y^** |
| --- | --- | --- | --- | --- | --- | --- | --- | --- | --- | --- | --- |
| **Patients** | **(Months)** | **Sex** | **FISH** | **IgVH** | **Positive (>30% )** | **Positive(>45%)** | **stage** | **Positive (>20% )** | **LD_50_^#^** | **% Axl** | **(at 0.5 µM)** |
| **P1** | 0 | M | 11q | unmutated | negative | negative | II | negative | ND | 60 |  |
|  | 3 |  | 11q | unmutated | negative | negative | IV | negative | >0.5 | 80.29 | 35.17 |
|  | 6 |  | 11q | unmutated | negative | negative | IV | negative | >0.5 | 90.11 | 29.84 |
|  | 9 |  | 11q | unmutated | negative | negative | IV | negative | >0.5 | 80.83 | 44.27 |
|  | 18 |  | 11q | unmutated | negative | negative | IV | negative | 0.144 | 88.82 |  |
|  | 23 |  | 11q | unmutated | negative | negative | IV | negative | 0.169 | 84.56 |  |
| **P2** | 0 | F | Tri-12 | unmutated | negative | positive | II | positive | ND | 71.5 |  |
|  | 2 |  | Tri-12 | unmutated | negative | positive | II | positive | >0.5 | 83.06 | 35.87 |
|  | 12 |  | Tri-12 | unmutated | negative | positive | II | positive | 0.125 | 83.26 |  |
|  | 21 |  | Tri-12 | unmutated | negative | positive | II | positive | 0.0813 | 65.06 |  |
| **P3** | 0 | M | Tri-12 | unmutated | positive | positive | 0 | positive | ND | 8.7 |  |
|  | 0.5 |  | 17p | unmutated | positive | positive | I | positive | 0.15 | 73.84 |  |
|  | 3 |  | 17p | unmutated | positive | positive | I | positive | 0.15 | 83.84 |  |
|  | 6 |  | 17p | unmutated | positive | positive | I | positive | 0.222 | 59.4 |  |
|  | 22 |  | 17p | unmutated | positive | positive | I | positive | 0.116 | 84 |  |
| **P4** | 0 | M | 11q | unmutated | positive | negative | I | positive | ND | 47.2 |  |
|  | 1 |  | 11q | unmutated | positive | negative | I | positive | 0.166 | 43.81 |  |
|  | 2 |  | 11q | unmutated | positive | negative | I | positive | ND | ND |  |
| **P5** | 0 | F | 17p | mutated | negative | negative | 0 | negative | ND | 4.8 |  |
|  | 8 |  | 17p | mutated | negative | negative | IV | negative | >0.5 | 20.4 | 5.53 |
|  | 12 |  | 17p | mutated | negative | negative | IV | negative | >0.5 | 20.59 | 10.96 |
|  | 14 |  | 17p | mutated | negative | negative | IV | negative | >0.5 | 4.66 | 6.95 |
|  | 16 |  | 17p | mutated | negative | negative | IV | negative | >0.5 | 7.45 | 8.44 |
|  | 22 |  | 17p | mutated | negative | negative | IV | negative | >0.5 | 6.5 | 6.33 |
|  | 23 |  | 17p | mutated | negative | negative | IV | negative | >0.5 | 16.61 | 4.87 |
|  | 27 |  | 17p | mutated | negative | negative | IV | negative | >0.5 | 18.54 | 10.75 |
|  | 30 |  | 17p | mutated | negative | negative | IV | negative | >0.5 | 15.74 | 14.38 |
| **P6** | 19 | M | 17p | unmutated | negative | positive | 0 | negative | ND | ND |  |
|  | 20 |  | 17p | unmutated | negative | positive | 0 | negative | ND | ND |  |
|  | 21 |  | 17p | unmutated | negative | positive | 0 | negative | 0.375 | 42.92 |  |
|  | 23 |  | 17p | unmutated | negative | positive | 0 | negative | 0.0875 | 40.48 |  |
| **P7** | 21 | F | 17p | unmutated | negative | positive | I | positive | 0.0875 | 89.66 |  |
| **P8** | 3 | M | 17p | unmutated | negative | negative | IV | positive | 0.175 | 30.1 |  |
|  | 7 |  | 17p | unmutated | negative | negative | IV | positive | 0.469 | 24.03 |  |
|  | 11 |  | 17p | unmutated | negative | negative | IV | positive | ND | ND |  |
| **P9** | 0 | M | Normal | mutated | positive | positive | 0 | negative | ND | 47.5 |  |
|  | 7 |  | Normal | mutated | negative | negative | IV | positive | 0.25 | 2.74 |  |
|  | 10 |  | Normal | mutated | negative | negative | IV | positive | 0.312 | 8.42 |  |
|  | 23 |  | Normal | mutated | negative | negative | IV | positive | 0.131 | 50.02 |  |
|  | 26 |  | Normal | mutated | negative | negative | IV | positive | 0.138 | 25.1 |  |
| **P10** | 0 | F | 11q | unmutated | positive | negative | III | positive | ND | 25.6 |  |
|  | 3 |  | 11q | unmutated | positive | negative | III | positive | 0.15 | 36.1 |  |
| **P11** | 5 | M | 13q | mutated | negative | negative | II | positive | 0.5 | 19.57 |  |
|  | 9 |  | 13q | mutated | negative | negative | II | positive | 0.47 | 26.73 |  |
| **P12** | 0 | F | 17p | mutated | negative | negative | III | negative | ND | 71 |  |
|  | 9 |  | 17p | mutated | negative | negative | III | negative | >0.5 | 67.47 | 35.55 |
|  | 14 |  | 17p | mutated | negative | negative | III | negative | 0.313 | 74.18 |  |
| **P13** | 0 | M | 11q | unmutated | positive | negative | I | negative | ND | 70.2 |  |
|  | 9 |  | 11q | unmutated | positive | negative | IV | positive | 0.3125 | 70.19 |  |
| **P14** | 0 | M | 13q | mutated | negative | positive | IV | positive | ND | 13.3 |  |
|  | 2 |  | 13q | mutated | negative | positive | IV | positive | >0.5 | 16.49 | 23.15 |
| **P15** | 0 | M | 11q | unmutated | negative | negative | I | positive | ND | 56.6 |  |
|  | 3 |  | 11q | unmutated | negative | negative | I | positive | 0.203 | 71.3 |  |
| **P16** | 0 | M | 13q | unmutated | positive | positive | IV | positive | ND | 25 |  |
|  | 9 |  | 13q | unmutated | positive | positive | IV | positive | 0.375 | 77.78 |  |
| **P17** | 0 | M | 13q | unmutated | negative | positive | IV | positive | ND | 40.6 |  |
|  | 9 |  | 13q | unmutated | negative | positive | IV | positive | >0.5 | 36.12 | 29.3 |
| **P18** | 0 | M | 13q | unmutated | negative | negative | I | positive | ND | 73.2 |  |
|  | 1 |  | 13q | unmutated | negative | negative | IV | positive | 0.438 | 87.61 |  |
| **P19** | 0 | M | Tri-12 | unmutated | positive | positive | I | positive | ND | 60.5 |  |
|  | 3 |  | Tri-12 | unmutated | positive | positive | I | positive | 0.203 | 85.71 |  |
| **P20** | 0 | M | 17p | mutated | positive | positive | 0 | negative | ND | 52.6 |  |
|  | 3 |  | 17p | mutated | positive | positive | 0 | negative | 0.33 | 91.25 |  |
|  | 19 |  | 17p | mutated | positive | positive | 0 | negative | 0.156 | 64.98 |  |
| **P21** | 0 | M | 13q | mutated | negative | negative | IV | negative | ND | 44.4 |  |
|  | 2 |  | 13q | mutated | negative | negative | IV | negative | 0.375 | 76.6 |  |
| **P22** | 0 | M | 13q | unmutated | negative | negative | 0 | negative | ND | 20.5 |  |
|  | 2 |  | 13q | unmutated | negative | negative | 0 | negative | >0.5 | 41.54 | 16.95 |
| **P23** | 0 | M | 13q | unmutated | negative | negative | II | negative | ND | 43.7 |  |
|  | 2 |  | 13q | unmutated | negative | negative | II | negative | 0.106 | 72.72 |  |
| **P24** | 16 | F | 17p | mutated | positive | negative | III | positive | 0.075 | 50.25 |  |
| **P25*** | 0 | F | 11q | unmutated | negative | negative | III | positive | 0.075 | 18.88 |  |
| **P26*** | 0 | M | 6q | mutated | negative | positive | III | positive | 0.075 | 49.13 |  |

“0” indicates time point before ibrutinib treatment started

“Ψ” % killing at 0.5 μM dose for patients whose LD_50_ is > 0.5 μM

“#” LD_50_ was determined from individual patient’s TP-0903 dose response curve

“*” Patients included as baseline of ibrutinib study

ND: Not done
